# Increased rate of FEV1 decline in HIV patients despite effective treatment with HAART

**DOI:** 10.1371/journal.pone.0224510

**Published:** 2019-10-29

**Authors:** Gloria Samperiz, Francisco Fanjul, Jose Luis Valera, Meritxell Lopez, Ángel Rios, María Peñaranda, Antoni Campins, Melchor Riera, Alvar Agusti

**Affiliations:** 1 Hospital Universitario Miguel Servet, Zaragoza, Spain; 2 Hospital Universitari Son Espases, Palma de Mallorca, Spain; 3 Institut d`Investigació Sanitària Illes Balears, Palma de Mallorca, Spain; 4 Respiratory Institute, Hospital Clinic, IDIBAPS, Univ. Barcelona, Barcelona, Spain; 5 CIBER Enfermedades Respiratorias, Palma de Mallorca, Spain; Northwestern University, UNITED STATES

## Abstract

**Introduction:**

Previous studies have reported that the rate of FEV1 decline over time is increased in HIV patients but the mechanisms underlying this observation are unclear. Since current HIV treatment with Highly Active Antiretroviral Therapy (HAART) results in very good immune-viral control, we hypothesized that HAART should normalize the elevated rate of FEV1 decline previously reported in HIV patients if it was somehow related to the immune alterations caused by HIV, particularly in never smokers or quitters, since smoking is a well established risk factor for accelerated FEV1 decline in the general population.

**Methods:**

We explored this hypothesis in a prospectively recruited cohort of 188 HIV (smoker and non-smoker) patients treated with HAART in Palma de Mallorca (Spain) and followed-up for 6 years. The cross-sectional characteristics of this cohort have been published elsewhere.

**Results:**

We found that: *(1)* HAART resulted in good immune-viral control; *(2)* the rate of FEV1 decline remained abnormally elevated, even in non-smokers and quitters; and, *(3)* alcohol abuse during follow-up was related to FEV1 decline in these patients.

**Discussion:**

Despite adequate immune-viral control by HAART, lung function decline remains increased in most HIV patients, even in non-smokers and quitters. Alcohol abuse is a preventable risk factor to decrease the accelerated FEV1 decline in this population.

## Introduction

The rate of lung function decline over time, as determined by changes in the expired volume in the 1^st^ second of a forced spirometry (FEV1), is increased in patients infected with the human immunodeficiency virus (HIV) [1–5]. The precise biological mechanisms underlying this observation are unclear but may include, among others, alterations in cellular immunity due to HIV infection, presence of chronic airway bacterial colonization due to a defective immune response and/or increased oxidative stress due to concurrent exposure to tobacco smoking or other toxic substances (marijuana, cocaine, heroin, alcohol) which are prevalent in this population [1–3], [6–13].

Highly Active Antiretroviral Therapy (HAART) results in very effective immune-viral control in patients infected with HIV [14]. We hypothesized that, if the enhanced rate of FEV1 decline previously reported in HIV patients was somehow related to the immune derangements caused by HIV, then effective HAART treatment (defined by a viral load < 200 copies/ml and/or CD4 counts higher than 350 cells/ml [15]) should normalize it, particularly in never smokers or quitters, since smoking is a well established risk factor for accelerated FEV1 decline in the general population. We explored this hypothesis in a prospectively recruited cohort of (smoker and non-smoker) HIV patients effectively treated with HAART, followed up in Palma de Mallorca (Spain) during 6 years. The cross-sectional characteristics of this cohort at recruitment have been published before [3].

## Methods

### Study design and ethics

**Fig 1:**
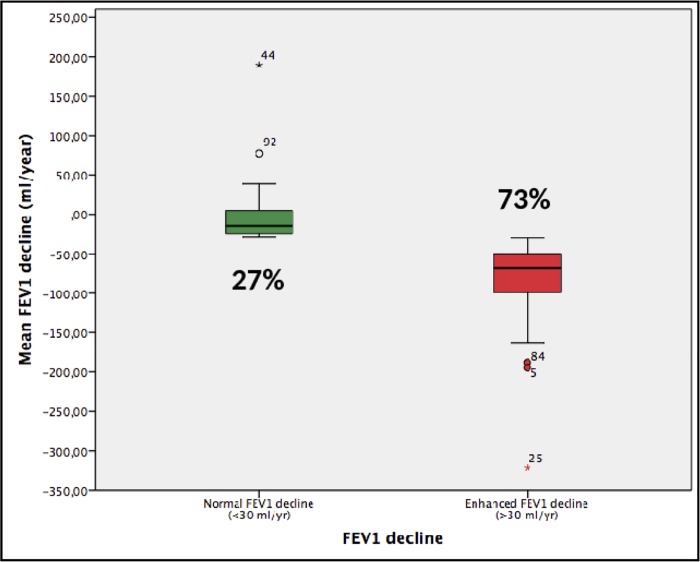
Box plot comparing the absolute FEV1 annual change in patients with normal (27%) or abnormal (73%) rate of FEV1 decline. For further explanations, see text.

Fig 1 presents the consort diagram of the study. Briefly, between July 2008 and March 2010, 285 HIV-infected patients signed their informed consent to participate in our cohort; 10 of them later refused to participate, so we finally included in our first cross-sectional analysis 275 patients [3]. These patients were followed-up regularly (4–6 months) in our clinic every six months until 2014–2016 (follow-up time 6.1±0.7 years), when those still attending the service were invited to be studied again. The study was approved by the Ethics Committee of the Balearic Islands, and all participants signed a new informed consent.

### Measurements

As reported before [3], symptoms and relevant clinical data were recorded using validated questionnaires (St. George’s Respiratory Questionnaire (SGRQ) [16] and Modified British Medical Research Council (mMRC) Questionnaire [17]). Toxic habits including tobacco and marijuana smoking and systemic drug use were self-reported. Alcohol consumption was measured with AUDIT test (AUDIT-C [18]). HIV predictors (viral load and quantification of lymphocyte populations) were determined in peripheral venous blood laboratories and equipment commonly used in our center. Forced spirometry and carbon monoxide lung diffusion capacity (DL_CO_) were measured (Medisoft Bodybox 5500, Medisoft, Belgium) following international guidelines [19]. Reference values were those of a Mediterranean population [20,21]. The presence (post-bronchodilator FEV_1_/FVC < 0.7) and severity of airflow limitation was established according to the GOLD criteria [1]. DL_CO_ was corrected for hemoglobin values following standard methodology. A DL_CO_< 80% of the reference value was considered abnormal [22]. The six-minute walking distance (6-MWD) was determined following international standards [23].

### Statistical analysis

Results are expressed as n (%), mean ± SD, or median [25–75 inter-quartile range], as appropriate. Unpaired T-test or Mann-Whitney U test were used to compare independent groups, whereas the McNemar test, Wilcoxson or paired T-test were used to compare groups at different time points. Correlations between continuous variables were explored by the Pearson or Spearman tests. Multivariable logistic regression analyses were used to explore the Odds Ratio (OR) of potential risk factors for FEV1 decline. A p value < 0.05 was considered significant. Statistical analyses were performed using SPSS version 22.0 (IBM, Armonk, NY).

## Results

### Characteristics of patients at recruitment

We included in this analysis 188 patients, 66% of those included in our original cohort, all of them with complete follow-up data (S1 Fig). As shown in S1 Table, there were no major demographic or clinical differences between them and those not included (n = 97;34%) in the current analysis.

Table 1 presents the main demographic and clinical characteristics of the 188 patients analyzed here. Most of them (76%) were males with a mean age of 49.2±6.7 years. Most participants (93%) were of Caucasian origin; the rest were of Latin-American (5%), Asian (1%), Caribbean (0.5%) or North-African (0.5%) origin. Table 1 also shows their toxic exposures at recruitment.

**Table 1:** Main characteristics (%, mean±SD or median [IQR]) of participants (n = 188) at recruitment and after 6 years follow-up.

	RECRUITMENT	FOLLOW-UP	P value
**Demographics**			
Males	76%		
Age. years	49.2±6.7	55.2±6.8	<0.001
BMI. kg/m^2^	24.0±3.8	24.5±4.0	0.002
**HIV data**			
Years with HIV+	13.3±5.3	19.2±5.1	<0.001
HAART	95.7%	98.4%	0.063
Undetectable viral load	93.6%	96.8%	0.210
Risk group			
Intravenous drugs	30.3%		
Homosexual	35.1%		
Heterosexual	31.9%		
Others	2.7%		
CDC AIDS phase			
A	38.3%		
B	30.9%		
C	30.9%		
CD4 nadir . cells mL^-1^	251 [108–468]		
CD4 nadir < 200 cell mL^-1^	77 (41.0%)		
CD4 <200 (month)	2 (0–18)		
Viral load zenit . copies/mL	44242 [6997.5–186181]		
CD4 cells mL^-1^	565.5 [394.5–814.75]	648.0 [466.5–888]	0.002
< 200 cell mL^-1^	6 (3.2%)	5 (2.7%)	0.004
200–350 cell mL^-1^	26 (13.8%)	18 (9.7%)	
350–500 cell mL^-1^	43 (22.9%)	32 (17.2%)	
>500 cell mL^-1^	113 (60.1%)	131 (70.4%)	
CD4/CD8	0.695 [0.44–1.045]	0.915 [0.6275–1.275]	<0.001
<0.6	74 (39.4%)	45 (24.2%)	<0.001
0.6–0.8	38 (20.2%)	33 (17.7%)	
>0.8	76 (40.4%)	108 (58.1%)	
**Toxic habits**			
Smoker			0.005
Never	15.4%	15.4%	
Former	28.2%	37.2%	
Current	56.4%	47.3%	
Pack-years	30.8±19	33.7±21	<0.001
Alcohol use	46.3%	70.2%	<0.001
Alcohol abuse	4.8%	19.7%	<0.001
Marijuana use	23.9%	22.3%	0.678
Cocaine use			0.001
Never	58.5%	51.1%	
Former	33.5%	43.1%	
Current	8.0%	5.8%	
Heroine use			1.000
Never	68.6%	68.6%	
Former	31.4%	31.4%	
Current	0.0%	0.0%	
**Previous diseases**			
*P*. *jiroveci* pneumonia	9.6%	9.6%	1.000
Hepatitis C	35.6%	35.6%	1.000
Tuberculosis	11.7%	11.7%	1.000
**Respiratory data**			
mMRC score. n (%)			<0.001
0	69.1%	74.8%	
1	26.6%	20.2%	
2	3.7%	3.1%	
3	0.5%	1.2%	
4	0.0%	0.6%	
SGRQ. median IQR)	6.7 [2.1–18.2]	7.8 [2.1–17.9]	0.334
FEV1 (postBD). % ref	94.6±13.9	90.2±14.7	<0.001
FVC (postBD). % ref	96.0±12.1	94.3±12.3	0.013
FEV1/FVC (postBD). %	77.7±8.5	74.1±9.0	<0.001

BMI: body mass index; SGRQ: St. George's Respiratory Questionnaire; mMRC: modified Medical Research Council breathlessness score; FEV_1_: forced expiratory volume in 1^st^ second; FVC: forced vital capacity.

HIV infection had been diagnosed 13.3±5.0 years before study entry. About a third of participants were in asymptomatic CDC stage (CDC-A), another third in stage CDC-B and the other third in CDC-C (the AIDS defining stage). Most of them (96%) were treated with HAART and had undetectable viral loads (<50 copies/mL); three patients were considered “elite controllers” (<50 copies/mL and CD4>500 cell ml^-1^) and were not treated with HAART. Median [IQR] CD4+ T cells value at recruitment was 565.5 [394.5–814.8] mL^-1^. Previous diagnosis of *P*. *jiroveci* pneumonia (9.6%), hepatitis C (35.6%) and tuberculosis (11.7%) were often reported.

Most participants were asymptomatic and had good health status (SGRQ). On average, spirometry was normal but, 17% of participants had evidence of airflow limitation, which was of mild (62.5%) or moderate (37.5%) severity. Only 5% of these patients had been previously diagnosed of COPD and only 30% of them received COPD treatment.

### Changes during follow-up

There was a significant reduction in current smoking habits and active cocaine consumption, but a significant rise in alcohol abuse (Table 1). HAART was indeed quite effective since only 6 patients (3.2%) presented a detectable viral load at the end of follow up and in all of them it was <200 copies/ml (91.2 [62.3–166.0] copies/ml).

In these well-controlled HIV patients, median FEV1 decline was 55 [28–87] ml/yr which is higher than the accepted upper limit of normal (30 ml/yr) [1,24]. As a result, the prevalence of airflow limitation increased from 17% at recruitment to 27% six year later (p<0.001), which at the end of follow up, was mild in 47% of patients, moderate in 51% and severe in 2%. To identify what variables were independently associated with FEV1 decline in this cohort, we used two complementary approaches.

First, we compared the clinical and physiological characteristics, both at recruitment and after six years follow-up, of patients with an FEV1 decline rate below or above the upper normal threshold value (30 ml/yr) [1,24] and used multivariable logistic regression analyses to explore the Odd Ratio (OR) of potential independent risk factors. Second, we considered changes in FEV1 during follow-up as a continuous variable and used lineal logistic regression analyses.

### Comparison of normal vs. rapid FEV1 decliners

Considering that the upper normal limit of FEV1 decline is 30 ml/yr [1,24], 27% of patients had a normal FEV1 decline during follow-up (median -14.6 [5.0/-24.0] ml/yr) whereas 73% showed increased FEV1 decline (median -67.8 [-49.7/-99.2] ml/yr, p<0.0001) (Fig 1). Patients with abnormal rate of FEV1 decline were more symptomatic (higher SGRQ score) and had lower FEV1 values at end of follow up (Table 2), albeit it was higher at recruitment. Of note, smoking exposure or status (never, former or current smoker) was not different in normal and rapid decliners. Finally, the occurrence of other respiratory diseases, viral load or circulating CD4 was not different between patients with normal or enhanced rate of FEV1 decline. Of note, however, alcohol abuse prevalence was higher in rapid decliners, both at recruitment and end of follow-up (Table 2). Multivariable analysis identified alcohol abuse and initial FEV1 values as independent factors associated with normal or abnormal FEV1 decline but excluded smoking exposure or current status at the end of follow-up (Table 3).

**Table 2:** Comparison of main demographic, clinical and lung function characteristics (%, mean±SD or median [IQR]) in patients with a normal (<30ml/yr) or increased (≥30ml/yr) rate of FEV1 decline, both at recruitment and end of six years follow-up. Among variables included in Table 1, only those with p value < 0.05 in the Bivariate analysis are shown.

	FEV1 decline < 30 ml/yr	FEV1 decline ≥30 ml/yr	p value
	n = 50 (27%)	n = 138 (73%)	
	-14.6 [5.0/-24.0] ml/yr	-67.8 [-49.7/-99.2] ml/yr	<0.001
**Toxic habits**			
Alcohol abuse Recruitment	0.0%	6.5%	0.017
Follow-up	10.0%	23.2%	0.044
**Respiratory data**			
SGRQ total score Recruitment	2.7 [1.7–13.9]	8.8 [3.5–19.1]	0.012
Follow-up	6.1 [0–16.9]	8.3 [3.3–18.3]	0.056
FEV1-postBD, % ref. Recruitment	90.0±14.2	96.3±13.5	0.006
Follow-up	95.2±13.5	88.4±14.8	0.005

SGRQ: St. George's Respiratory Questionnaire.

**Table 3:** Univariate and multivariable analysis of predictive factors of increased rate (≥30 ml/yr) of FEV1 decline in HIV patients. Among variables included in Table 1, only those with p value < 0.05 in the Bivariate analysis are shown.

	Univariate analysis			Multivariable analysis		
	Unadjusted OR	CI 95%	P value	Adjusted OR	CI 95%	P value
**Toxic habits**						
Alcohol abuse follow-up	2.717	0.995–7.422	0.051	3.1883	1.049–9.683	0.041
**Respiratory data**						
FEV1-postBD, % ref. recruitment	1.035	1.007–1.064	0.016	1.030	1.005–1.056	0.019

### Variables associated with absolute FEV1 changes during follow-up

Table 4 presents the list of factors associated with FEV1 decline (when it was considered a continuous variable and not dichotomized into normal and rapid decliners (see below)). Smoking exposure, alcohol abuse during follow-up, years of HIV infection and forced vital capacity at recruitment were identified as independent risk factors of FEV1 decline in this cohort.

**Table 4:** Predictors of FEV1 decline (considered as a continuous variable). Among variables included in Table 1, only those with p value < 0.10 in the Bivariate analysis are shown.

	Bivariate (Spearman) Rho/P value		Multivariable Coefficient B(CI(95%)/P value	
**Demographics**				
BMI (Kg/m^2^)	-0,206	0.005		
**Toxic habits**				
Smoker recruitment	-0.178	0.015		
Pack-years recruitment	-0.177	0.026		
Pack-years follow-up	-0.199	0.015	-0.453 (-0.112/-0,793)	0.010
Alcohol abuse follow-up	-0.174	0.017	-20.367 (-2.706/-38.028)	0.024
**HIV data**				
Years with HIV+	-0.125	0.087	-1.53 (-0.156/-2.903)	0.029
**Respiratory data**				
SGRQ total score recruitment	-0.195	0.008		
SGRQ total score follow-up	-0.195	0.007		
FVC-postBD (% ref) recruitment	-0.270	<0.001	-0.951 (-0.325/-1.576)	0.003
FEV1-postBD (%) recruitment	-0.240	0.001		
FVC-postBD (% ref) follow-up	-0.214	0.003		
FEV1-postBD (% ref) follow-up	-0.279	<0.001		
FEV1/FVC (%) follow-up	-0.177	0.015		

BMI: body mass index; SGRQ: St. George's Respiratory Questionnaire; FVC: forced vital capacity.

### Effects of smoking

**Fig 2:**
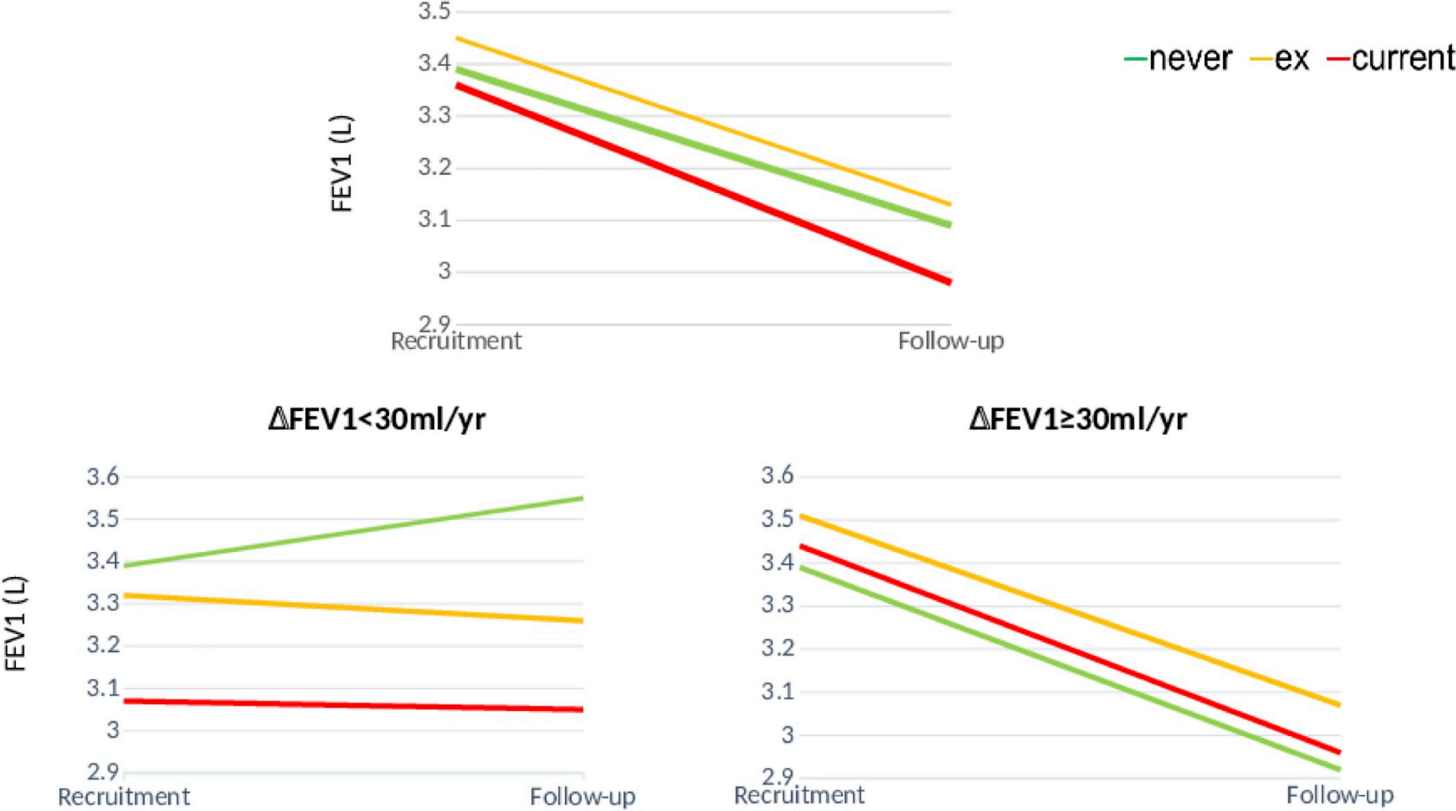
FEV1 trajectories from baseline to end of follow-up in participants with normal (left) or accelerated decliners in addition to the FEV1 changes (right) stratified by smoking status at the end of follow up (never (green line), former (yellow line) and current smokers (red line). The annualized rates of FEV1 decline within each group were not significantly different. For further explanations, see text.

At the end of follow-up 52.6% of participants were either never (15.4%) or former smokers (37.2%), whereas 47.3% of them were still active smokers. Table 5 presents the variables that were significantly different between these two groups at the end of follow-up. Main observations were that, in current smokers, the prevalence of other toxic exposures (alcohol, marijuana, cocaine and heroin) was higher, some HIV related variables were different, the prevalence of comorbidities was surprisingly lower and, not surprisingly, they had more respiratory symptoms and worse lung function. Of note, their FEV1 decline and the proportion of patients with abnormal FEV1 decline tended to be higher but differences did not achieve statistical significance (Table 5). In keeping with this, Fig 2 shows that the mean FEV1 trajectories from baseline to the end of follow-up in participants with normal (left panel) or enhanced (right panel) rates of FEV1 decline were not influenced by smoking status at the end of follow up (never (green line), former (yellow line) and current smokers (red line). Of note, within each group (normal and abnormal FEV1 decline) were not significantly different.

**Table 5:** Statistically significant differences between current vs. never and former smokers at the end of follow-up. Variables included in Table 1 not shown here were not significantly different between these two groups at this time point.

	CURRENT SMOKERS	NEVER AND FORMER SMOKERS	P value
	n = 89 (47.3%)	n = 99 (52.7%)	
**Demographic characteristics& toxic exposures**			
Age in years. mean (SD)	53.3±5.1	57.0±7.7	<0.001
BMI in kg/m^2^	23.4±3.5	25.5±4.2	<0.001
Pack-years	36 [27–46]	25 [15–35]	0.002
Alcohol abuse	23 (25.8%)	14 (14.1%)	0.044
Marijuana use	34 (38.2%)	8 (8.1%)	<0.001
Cocaine use			0.003
Never	33 (37.1%)	63 (63.6%)	
Former	51 (57.3%)	30 (30.3%)	
Current	5 (5.6%)	6 (6.1%)	
Heroine use			<0.001
Never	50 (56.2%)	79 (79.8%)	
Former	39 (43.8%)	20 (20.2%)	
Current	0 (0.0%)	0 (0.0%)	
**HIV Data**			
HIV Risk group. n (%)			0.003
Intravenous drugs	37 (41.6%)	20 (20.2%)	
Homosexual	27 (30.3%)	39 (39.4%)	
Heterosexual	24 (27.0%)	36 (36.4%)	
Others	1 (1.1%)	4 (4.0%)	
Years with HIV+. mean (SD)	20.1±5.4	18.1±4.7	0.007
CD4 nadir. median (IQR)	301 [187–528]	194 [65–422]	0.003
CD4. median (IQR)	728 [516–912]	598 [429–823]	0.018
CD4 <200 (MONTH)	0 [0–12]	6 [0–20]	0.002
CD4 nadir < 200	24 (27.0%)	53 (53.5%)	<0.001
Hepatitis C. n (%)	42 (47.2%)	25 (25.3%)	0.002
**Comorbidities**			
Diabetes. n (%)	6 (6.7%)	16 (16.2%)	0.045
HTA. n (%)	23 (25.8%)	44 (44.4%)	0.008
**Respiratory Symptoms & Lung Function**			
SGRQ. median (IQR) Total	12.6 [5.5–20.9]	4.7 [0.9–10.8]	<0.001
FEV1-postBD (%). mean (SD)	87.8±15.4	92.4±13.8	0.030
FEV1/FVC postBD. mean (SD)	71.4±9.19	76.6±8.2	<0.001
DLCO (%). mean (SD)	69.5±12.9	80.1±14.9	<0.001
DLCO <80%	68 (76.4%)	45 (45.5%)	<0.001
WT6M (%). mean (SD)	93.32±10.38	104.0±31.3	0.004
FEV1 decline (ml), median (IQR)	-61.3 [-97.6;-32.7]	-47.8 [-82.7;-25.6]	0.088
FEV1decline > 30 ml/year, n (%)	69 (77.5%)	69 (69.7%)	0.225

## Discussion

The two main findings of this prospective study in HIV patients with good immune-viral control by HAART were that: *(1)* FEV1 decline remained increased in about three quarters of them; and, *(2)* smoking exposure/status does not seem to play a key pathogenic role, but alcohol abuse emerges as an important preventable risk factor.

### Previous studies

In the pre-HAART era, the HIV Lung Complications study showed that FEV1 decline was enhanced in HIV-infected patients followed-up for about 4 years [25]. In the HAART era, the ALIVE study showed that poorly controlled HIV disease was indeed associated with accelerated lung function decline [26]. Three years later, Kunisaki *et al* showed that the timing of HAART initiation did not influence the rate of lung function decline in HIV-positive individuals who were naive to treatment and had CD4+ T-cell counts higher than 500 per μL [27]. A few months ago, Ronit *et al* confirmed that HIV infection is indeed associated with low lung function values and, interestingly, in keeping with our observations here, that this is not explained by smoking or socioeconomic status and may be mediated by prior immunodeficiency [28]. Our study extends these previous observations by showing that, even in HIV patients with good immune-viral control due to effective HAART, FEV1 decline is still higher than normal in the majority (73%) of individuals, including never or former smokers, and identifies novel and preventable mechanisms underlying this observation, such as alcohol abuse.

### Interpretation of novel findings

In theory, the high rate of FEV1 decline observed in our HIV patients treated with HAART can be attributed to one or more of the following factors: *(1) immune alterations related to HIV infection* [6–8]. At first glance, the fact that FEV1 decline in our cohort was higher than normal despite good immune-viral control may suggest that immune alterations related to HIV infection are not a main mechanistic driver. However, there are some subtle caveats that need careful consideration here. On the one hand, we did not assess directly the potential pulmonary immune derangements associated to HIV and HAART in these patients so we cannot exclude that immune-senescence or partial recovery of immunity may still contribute to the elevated rate of FEV1 decline observed in our patients [29]. In this context, it is of note that years of HIV infection were indeed identified as significant as a risk factor of FEV1 decline (Table 3). On the other hand, though, CD4 nadir has been previously associated with emphysema and pulmonary function in people living with HIV [7,28,30], but we were unable to identify significant differences in FEV1 decline between patients with a CD4 nadir lower or higher than 200 cell/ml; *(2) exposure to tobacco smoking*. We found that tobacco smoking was a significant risk factor for FEV1 decline when it was considered as a continuous variable but not when patients were dichotomized into normal and rapid decliners (Fig 2). This is in keeping with the recent observations by Ronit *et al* who also reported no interaction between HIV and cumulative smoking [28]. Needless to say, that this should not detract from the importance of encouraging quitting smoking in the clinic; *(3) respiratory infection* [9–11, 31]. We did not observe any significant difference in the previous history of respiratory infections between patients with normal or accelerated FEV1 decline, suggesting that they were not major patho-biological players either in this scenario; *(4)*, unexpectedly, we observed that alcohol abuse was an independent and significant risk factor for the high FEV1 decline seen in this population, both when FEV1 changes were considered in the analysis as a continuous or dichotomized variable. The relationship between alcohol abuse and lung function is not well characterized but there is evidence that it impairs alveolar epithelial surfactant production and barrier integrity, decreases alveolar macrophage function, and renders the lung susceptible to oxidant-mediated injury [32]. In fact, epidemiological studies have shown that heavy drinkers have more symptoms of chronic bronchitis than non-heavy drinkers, even after adjusting for smoking exposure, suggesting that heavy drinking has an independent additive negative effect on lung function in smokers [33]; and, finally *(5)* it is of note also that a better initial lung function was also independently associated with a higher rate of FEV1 decline (Table 2). This is known as “the horse-racing effect of lung function” [34], and has been well described in other non-infected HIV populations, where those with higher initial FEV1 values are those who lose more during follow-up [35,36].

### Clinical implications

Our study is purely observational and does not include any therapeutic intervention, so it does not have direct clinical implications. However, we would like to speculate with some potential implications for clinical practice. First, given the high prevalence and incidence of respiratory abnormalities observed in HIV infected patients despite being effectively treated with HAART (Fig 1), we suggest that lung function should be monitored in the clinic routinely in order to offer these patients specific respiratory treatment if needed. And, secondly, the abandonment of smoking habits and the abuse of other drugs in this population (as, no doubt, in any other) should be reinforced. However, given the results discussed above, alcohol abuse deserves particular therapeutic attention in HIV-infected patients.

### Potential limitations

A potential limitation of our study is that we did not include a control group. Yet, what this control group should be is not straightforward. The ideal comparator would be a group of untreated HIV infected patients. Obviously, this is not possible because it would be unethical to withhold treatment to these patients. An alternative can be the use of uninfected individuals as controls, as recently reported by Ronit *et al* [28]. Strictly speaking, however, this strategy compares two different groups of individuals (infected vs. healthy). A third alternative, which is the one we used here, is to compare our results in current and former/never smokers as well as to contrast them with those previously reported in the literature in the general population.

Another limitation could be to make the study in a single-center, and there may be environmental changes that affect the decline of these patients, and cannot be verified in an uninfected population. Also, one potential bias in our study is due to missing data. After checking the database, we found that data was only missing from the outcome variable so we decided to use a complete case analysis following recent evidence.

## Conclusions

Despite adequate immune-viral control by HAART, the rate of FEV1 decline remains elevated in a majority of HIV patients. Smoking does not appear to be the main driver of this observation but alcohol abuse emerges as a novel, important, and preventable, risk factor.

## Supporting information

S1 Fig Consort diagram of the study. (TIFF)

S1 Table Comparison of the main demographic and clinical characteristics (n (%) or mean ± SD) of participants who completed the study (n = 188) vs. those lost for follow-up (n = 97). (DOCX)
