# Supplementary figures and images for: Increased rate of FEV1 decline in HIV patients despite effective treatment with HAART
Source: PLoS One. 2019 Oct 29;14(10):e0224510. doi: 10.1371/journal.pone.0224510 (PMC6818778; doi:10.1371/journal.pone.0224510)

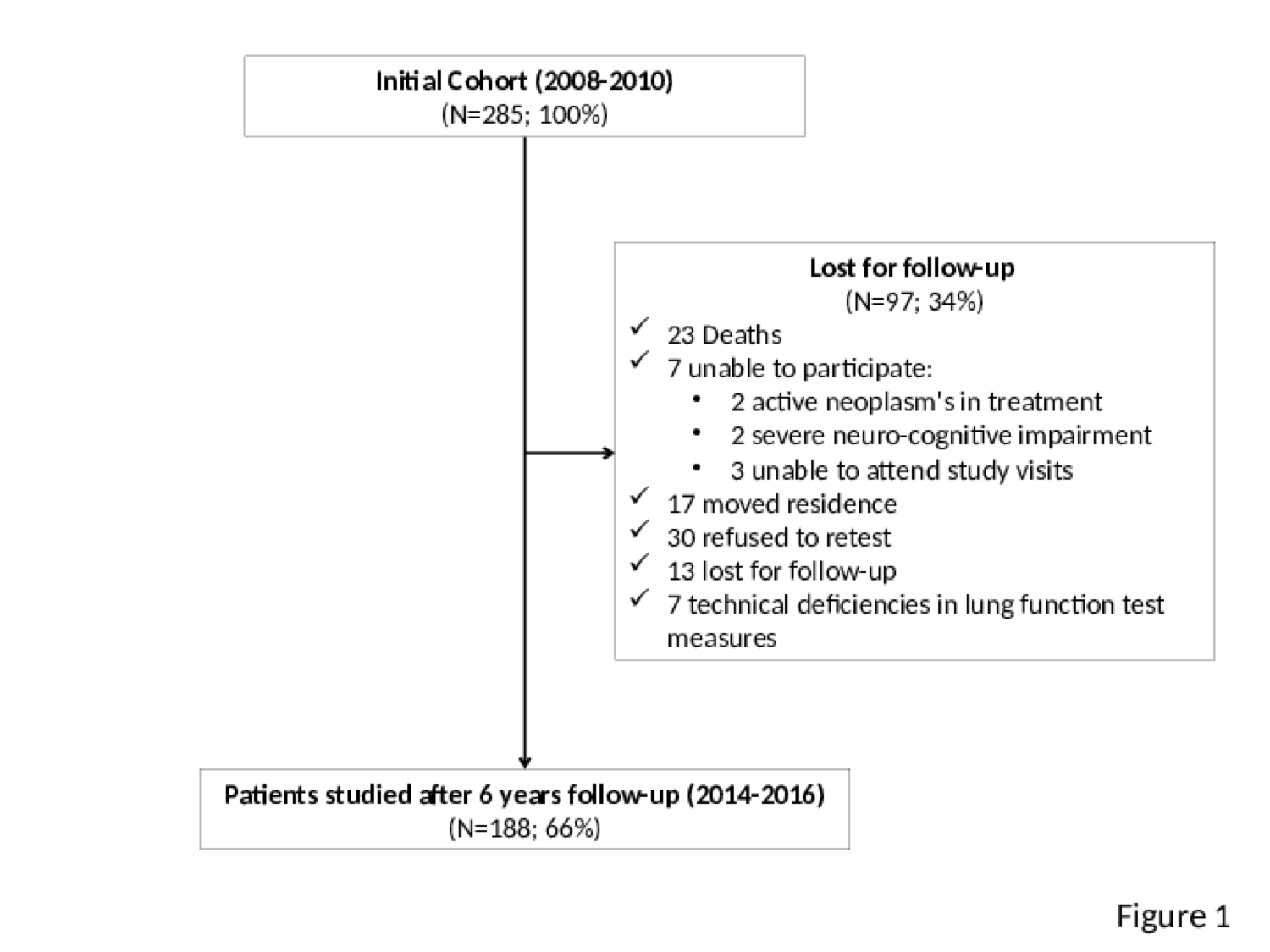

Supplement: S1 Fig — (TIFF) [file pone.0224510.s001.tiff]
