# Supplementary material for: Increased rate of FEV1 decline in HIV patients despite effective treatment with HAART
Source: PLoS One. 2019 Oct 29;14(10):e0224510. doi: 10.1371/journal.pone.0224510 (PMC6818778; doi:10.1371/journal.pone.0224510)
Supplement: S1 Table — (DOCX) [file pone.0224510.s002.docx]

Friday, 30 November 2018

ON-LINE SUPPLEMENT

**INCREASED rate of fev1 DECLINE IN HIV PATIENS despite effective treatment with haart**

Samperiz G^1^, Fanjul F^2,3^, Valera JL^2^, Lopez M^3^, Rios A^3^,

Peñaranda M^2,3^, Campins A^2,3^, Riera M^2,3^, Agusti A^4,5^

(1) Hospital Universitario Miguel Servet, Zaragoza, Spain

(2) Hospital Universitari Son Espases, Palma de Mallorca, Spain

(3) Institut d`Investigació Sanitària Illes Balears, Palma de Mallorca, Spain

(4) Respiratory Institute, Hospital Clinic, IDIBAPS, Univ. Barcelona, Spain

(5) CIBER Enfermedades Respiratorias, Spain.

**On-line supplement,** Tables: 1. Figures: 1

**Table S1.** Comparison of the main demographic and clinical characteristics (n (%) or mean ± SD) of participants who completed the study (n=188) vs. those lost for follow-up (n=97).

|  | **Patients with complete follow up data**  **(N=188)** | | **Patients lost during follow up**  **(N= 97)** | | **p value** |
| --- | --- | --- | --- | --- | --- |
| **Demographics** | | | | | |
| Males | 143 | 76% | 79 | 81% | 0.300 |
| Age, years | 48.6±6.7 | | 48.3±6.3 | | 0.689 |
| BMI, Kg/m2 | 24.0 ±3.8 | | 24.1 ±5.6 | | 0.790 |
| **Toxic habits** | | | | | |
| Smoking exposure, n (%) |  |  |  |  | 0.027 |
| Never | 29 (15%) | | 9 (10%) | |  |
| Former | 53 (28%) | | 19 (20%) | |  |
| Current | 106 (56%) | | 67 (71%) | |  |
| Pack-years | 30.8±19.2 | | 30.5±18.5 | | 0.903 |
| Cannabis, n (%) |  |  |  |  | 0.413 |
| No | 143 (76%) | | 68 (72%) | |  |
| Yes | 45 (24%) | | 27 (28%) | |  |
| Alcohol use, n (%) |  |  |  |  | 0.111 |
| Never | 66 (35%) | | 25 (26%) | |  |
| Former | 36 (19%) | | 18 (19%) | |  |
| Current | 86 (46%) | | 52 (55%) | |  |
| Cocaine use, n (%) |  |  |  |  | 0.250 |
| Never | 110 (59%) | | 49 (52%) | |  |
| Former | 63 (34%) | | 36 (38%) | |  |
| Current | 15 (8%) | | 10 (11%) | |  |
| Heroine use, n (%) |  |  |  |  | 0.835 |
| Never | 129 (69%) | | 65 (68%) | |  |
| Former | 59 (31%) | | 29 (31%) | |  |
| Current | 0 (0%) | | 1 (1%) | |  |
| **HIV data** | | | | | |
| Risk group, n (%) |  |  |  |  | 0.600 |
| Intravenous drugs | 57 (30%) | | 31 (33%) | |  |
| Homosexual | 66 (35%) | | 31 (33%) | |  |
| Heterosexual | 60 (32%) | | 32 (34%) | |  |
| Blood transfusions | 1 (1%) | | 1 (1%) | |  |
| Others | 4 (2%) | | 0 (0%) | |  |
| CDC AIDS phase, n (%) |  |  |  |  | 0.249 |
| A | 72 (38%) | | 47 (49%) | |  |
| B | 58 (31%) | | 22 (23%) | |  |
| C | 58 (31%) | | 28 (29%) | |  |
| Years with HIV+, median [IQR] | 13.1 [8.58-16.56] | | 13.5 [8.4-16.93] | | 0.859 |
| CD4 <200 cells mL^-1^ (month) | 2 [0-18] | | 3 [0-15] | | 0.723 |
| CD4 nadir , cells mL^-1^ | 247 [107.5-462] | | 296.5 [137.5-485] | | 0.339 |
| Viral load zenith , (log) | 4.65 [3.86-5.26] | | 4.75  3.93-5.4 | | 0.460 |
| CD4 cells mL^-1^ | 565.5 [395-814.5] | | 498 [389-767] | | 0.302 |
| CD4/CD8 | 0.7 [0.44-1.04] | | 0.65 0.43-1.00] | | 0.438 |
| Undetectable viral load | 176 (94%) | | 83 (88%) | | 0.124 |
| HAART | 180 (96%) | | 91 (96%) | | 0.986 |
| **Previous diseases** | | | | | |
| Tuberculosis, n (%) | 22 (12%) | | 9 (10%) | | 0.571 |
| *P. jiroveci* pneumonia | 18 (10%) | | 9 (10%) | | 1.000 |
| Hepatitis C, n (%) | 67 (36%) | | 35 (37%) | | 0.793 |
| **Respiratory data** | | | | | |
| SGRQ total | 6.7 [2.1-18.2] | | 8.7 [2.7-21.4] | | 0.341 |
| mMRC score, n (%) |  |  |  |  | 0.210 |
| 0 | 130 (69%) | | 56 (60%) | |  |
| 1 | 50 (27%) | | 29 (31%) | |  |
| 2 | 7 (4%) | | 7 (7%) | |  |
| 3 | 1 (1%) | | 2 (2%) | |  |
| 4 | 0 (0%) | | 0 (0%) | |  |
| FVC-postBD (% ref) | 96.5 [88.5-103.6] | | 90.9 [(80.1-100.7)] | | 0.004 |
| FEV1-postBD (% ref) | 95.9 [86.6-103.3] | | 92.6 [76.1-102.5] | | 0.054 |
| FEV1/FVC (%) | 79.2 73.5-84.1`+ | | 79.1 [ (68.0-84.3] | | 0.353 |

BMI: body mass index; SGRQ: St. George's Respiratory Questionnaire, mMRC: modified Medical Research Council breathlessness score. FEV_1_: forced expiratory volume in 1^st^ second; FVC: forced vital capacity.

**Figure S1.** Consort diagram of the study.
